# Genome-Wide Association Mapping of Major Root Length QTLs Under PEG Induced Water Stress in Wheat

**DOI:** 10.3389/fpls.2018.01759

**Published:** 2018-11-29

**Authors:** Habtamu Ayalew, Hui Liu, Andreas Börner, Borislav Kobiljski, Chunji Liu, Guijun Yan

**Affiliations:** ^1^School of Agriculture and Environment, Faculty of Science, The UWA Institute of Agriculture, The University of Western Australia, Crawley, WA, Australia; ^2^Noble Research Institute LLC, Ardmore, OK, United States; ^3^Genebank Department, Leibniz Institute of Plant Genetics and Crop Plant Research (IPK), Gatersleben, Germany; ^4^Institute of Field and Vegetable Crops, Novi Sad, Serbia; ^5^CSIRO Agriculture Flagship, Townsville, QLD, Australia

**Keywords:** genome wide association, hexaploid wheat, linkage disequilibrium, root length, water stress

## Abstract

Roots are vital plant organs that determine adaptation to various soil conditions. The present study evaluated a core winter wheat collection for rooting depth under PEG induced early stage water stress and non-stress growing conditions. Analysis of phenotypic data indicated highly significant (*p* < 0.01) variation among genotypes. Broad sense heritability of 59 and 73% with corresponding genetic gains of 7.6 and 9.7 (5% selection intensity) were found under non-stress and stress conditions, respectively. The test genotypes were grouped in to three distinct clusters using unweighted pair group method with arithmetic mean (UPGMA) clustering based on maximum Euclidian distance. The first three principal components gave optimum mixed linear model for genome wide association study (GWAS). Linkage disequilibrium (LD) analysis showed significant LD (*p* < 0.05) amongst 15% of total marker pairs (25,125). Nearly 16% of the significant LDs were among inter chromosomal marker pairs. GWAS revealed five significant root length QTLs spread across four chromosomes. None of the identified QTLs were common between the two growing conditions. Stress specific QTLs, combined explaining 31% of phenotypic variation were located on chromosomes 2B (*wPt6278*) and 3B (*wPt1159*). Similarly, two of the three QTLs (*wPt0021* and *wPt8890*) identified under the non-stress condition were found on chromosomes 3B and 5B, respectively. The B genome showed significant importance in controlling root growth both under stress and non-stress conditions. The identified markers can potentially be validated and used for marker assisted selection.

## Introduction

Common wheat (*Triticum aestivum* L.) is one of the earliest cereals ever domesticated and is currently one of the major sources of food and feed in the world. Wheat is adapted to diverse climatic zones including drought prone areas (Tardif et al., 2007; Monneveux et al., 2012). Changes in global climate and expansion of wheat production to less optimum production zones are causing severe cop losses annually (Olmstead and Rhode, 2010; Tanaka et al., 2015). Water stress is one of the grand challenges limiting crop growth and productivity in various parts of the world (Fleury et al., 2010; Zhao and Dai, 2015). The unabated expansion of global warming and erratic rainfall pattern remain to be threats for global food security (Wheeler and Von Braun, 2013).

Crop productivity in dry areas can be improved through appropriate exploitation of available genetic variability of crop plants to better adapt to climate change (Pieruschka and Lawson, 2015; Reynolds and Langridge, 2016). Reintroducing valuable alleles from wild progenitors of crop plants helps enrich domesticated gene pool (Feldman and Millet, 2001; Gur and Zamir, 2004). In this regard, wild emmer (*Triticum turgidum*) has been reported to harbor rich allelic diversity for numerous traits, including deep rooting for water stress resistance (Peng et al., 2012; Merchuk-Ovnat et al., 2017).

Water stress resistance in plants involves intricate physiochemical pathways ranging from cellular to whole-plant signaling (Tardieu, 2012, 2016; Janiak et al., 2015). Therefore, it is necessary to break down genetic analysis into smaller scales including cells and organs to better understand the underlying genetic mechanisms of water stress resistance. Plant structural traits including deep rooting, thick wax layer, spiny leaves, and acute leaf angle are frequently investigated due to their role in water stress resistance (Wasson et al., 2012; Comas et al., 2013). Deep rooting is an important root architectural trait that enables access water from deeper soil profiles thereby improving crop productivity. Gao and Lynch (2016) demonstrated that deeper roots in maize improved water acquisition, and as a result, biomass and grain yield. Similarly, deep rooting has been reported to improve grain yield in rice (Uga et al., 2013) and chickpea (Varshney et al., 2013). However, phenotyping roots on a large number of genotypes is time taking and labor intensive. As a result, plant roots are less explored compared with above ground parts (Fleury et al., 2010). Employing molecular markers to run foreground and background germplasm screening helps minimize labor and time required to phenotype roots thereby improving selection efficiency (Varshney et al., 2009; Beyene et al., 2016; Rai et al., 2018). Deep rooting QTLs have been identified and closest markers have been validated for marker assisted breeding in wheat (Ayalew et al., 2017), and rice (Obara et al., 2010; Uga et al., 2013). However, most previous studies used bi-parental structured populations, which are not effective in exploiting available allele diversity in the gene pool. Genome wide association studies (GWAS) on the other hand is an innovative approach to accommodate as many allelic diversity as possible.

Gaut and Long (2003), Meuwissen and Goddard (2004), Bradbury et al. (2007), Kang et al. (2008), Ramya et al. (2010), Zhang et al. (2010), Lipka et al. (2012), Qie et al. (2014), Tadesse et al. (2015), Ayalew et al. (2018), Onyemaobi et al. (2018). Compared with above ground plant parts, genome wide association studies on root growth are limited. This study was conducted to (1) characterize genotypic and phenotypic diversity of a core winter wheat collection, (2) analyze LD and population structure, and (3) identify genomic regions significantly associated with root length under water stress and non-water stress growing conditions.

## Materials and Methods

### Plant Materials and Phenotypic Evaluation

Ninety-one genotypes of a winter wheat core collection obtained from the Institute of Field and Vegetable Crops (Novi Sad, Serbia) (Table 1) were evaluated for root length at early plant growth stage both under water stress and non-stress conditions. The core collection consists of diverse genotypes which were collected from 21 countries across five continents (Neumann et al., 2011). Phenotypic evaluation for osmotic stress was carried out in a controlled growing environment at the school of plant biology, The University of Western Australia. A hydroponic culture optimized for a similar research by Ayalew et al. (2015) was used. Plastic boxes of 3,000 ml were used with 8 mm diameter holes drilled on lids. The tops of the lids were lined with filter paper to keep plants in place and the surface moist. Seeds were first germinated in Petri dishes lined with filter paper for 48 h and then healthy and vigorous seedlings were transferred to the water system organized in a randomized complete block design with three replicates. Each replication was represented by mean value of two individual plants. Osmotic stress of −0.5 MPa was induced using PEG 6000 (Sinopharm Chemical Reagent Co., Ltd., Shanghi, China). The final stress level during data collection was measured using MP4 dewpoint potentiameter (Decagon Devices Inc., 2003) and the stress was progressive which reached −0.6 ± 0.1 MPa at the last date of the stress period. Plant nutrition in the form of half strength Hoagland’s solution, and water stress (-0.5 MPa) using PEG6000 solution were added 7 days after germination for the treatment set (stressed), and Hoagland’s solution alone for the control (non-stressed) set, respectively. The pH of the solution was adjusted to 5.5–5.7 while relative humidity was between 65 and 70%. The temperature was set to 25/22°C day/night while light intensity of 300 μmol.m^−2^.s^−1^ was supplied using cool florescent lamps in 10/14 h dark and light timing. The solution was being constantly aerated by bubbling air in to the solution using an electric bubbler. Data were scored on root length 17 days after planting. Graduated ruler (cm) was used to measure the length of the longest roots in each replicate sample.

**Table 1:** Genotypes (91) used in this experiment and their respective countries of origin.

Accession name	Origin	Accession name	Origin	Accession name	Origin
Magnif 41	Argentina	Acciaio	Italy	PKB Krupna	Serbia
Gala	Argentina	Ai-bian	Japan	NS 46/90	Serbia
Kite	Australia	Norin 10	Japan	Mina	Serbia
Minister Dwarf	Australia	Saitama - 27	Japan	NS 63-24	Serbia
Mexico 120	Australia	Tr. Compactum	Latvia	NS 74/95	Serbia
Timson	Australia	Vireo “S”	Mexico	NS 79/90	Serbia
Triple dirk “S”	Australia	Mex. 3	Mexico	Avalon	United Kingdom
Tr. dirk “B”(GK 775)	Australia	Cajeme 71	Mexico	Brigand	United Kingdom
Cook	Australia	Siete Cerros	Mexico	TJB 990-15	United Kingdom
Tr. dirk “B”(GK 12)	Australia	Inia 66	Mexico	Highbury	United Kingdom
Rusalka	Bulgaria	Mex. 17 bb	Mexico	Mironovska 808	Ukrain
Lambriego Inia	Chile	BCD 1302/83	Moldova	HAYS 2	United States
Ching-Chang 6	China	F 4 4687	Romania	WWMCB 2	United States
Al Kan Tzao	China	Donska polup.	Rusia	INTRO 615	United States
Peking 11	China	Bezostaya 1	Rusia	UC 65680	United States
Ana	China	NS 602	Serbia	Vel - USA	United States
ZG 987/3	China	NS 559	Serbia	Semilia Eligulata	United States
ZGK 238/82	China	L 1A/91	Serbia	Holly E	United States
ZG 1011	China	L 1/91	Serbia	Centurk	United States
Tibet Dwarf	China	NS 33/90	Serbia	Helios	United States
Tom Thumb	China	Sofija	Serbia	Florida	United States
Durin	France	Nizija	Serbia	Tr.Sphaerococcum	United States
Capelle Desprez	France	Sava	Serbia	Benni multifloret	United States
L-1	Hungary	NS 55-25	Serbia	Hope	United States
Szegedi 768	Hungary	Slavija	Serbia	Norin10/Brevor14	United States
Bankuty 1205	Hungary	Nov. Crvena	Serbia	Phoenix	United States
Hira	India	Pobeda	Serbia	Lr 10	United States
UPI-301	India	Renesansa	Serbia	Purd./Loras	United States
Sonalika	India	Ivanka	Serbia	Red Coat	United States
Suwwon 92	India	NS 22/92	Serbia	Purdue 39120	United States
				Purdue 5392	United States

### Molecular Marker Data

The test genotypes were previously assayed using diversity array technology (DArT) markers by Triticarte Pty. Ltd. (Canberra, ACT, Australia^1^), a whole-genome profiling service laboratory, as described by Neumann et al. (2011). Five hundred and thirty-three polymorphic DArT markers with known linkage positions, based on CIMMYT integrated map (Crossa et al., 2007) were used (Supplementary Figure S1). Average *p*-value, call rate and polymorphism information content (PIC) of all of the markers were 86, 0.35, and 95, respectively.

### Data Analysis

#### Phenotypic Data Analysis

Phenotypic data were subjected to statistical analysis using CropStat 2007.3 (International Rice Research Institute, 2007) software accounting for measurement and block effects based on the following fixed effects model: y_ij_ = μ + g_i_ + b_j_ + ε_ij_, where y_ij_ is the observed mean, μ is the general mean, g_i_ is the genotype, b_j_ is the block and ε_ij_ is the error effects. Variance components were estimated as: $\delta_{\text{g}}^{2}=\frac{\left(\mathrm{MSg}-\mathrm{MSe}\right)}{r}$ while $\delta_{\text{e}}^{2}=\frac{\mathrm{MSe}}{r}$, where MSg is mean square of the lines, MSe is the residual error and r the number of replicates and broad-sense heritability were estimated using the following expression: H^2^ = $\delta_{\text{g}}^{2}/(\delta_{\text{g}}^{2}+\delta_{\left(\text{e}/\text{r}\right)}^{2})$, where $\delta_{\text{g}}^{2}$ and $\delta_{\text{e}}^{2}$ are the estimated genotypic and error variances, respectively (Nyquist and Baker, 1991). Genetic gain was calculated using the formula: Gs = K^∗^H^2∗^($\delta_{\text{p}}^{2}$)^−1/2^, where K is the selection intensity at 5% (*k* = 2.056), H^2^ is heritability in broad sense and, ($\delta_{\text{p}}^{2}$)^−1/2^ is phenotypic standard deviation.

#### Population Structure and Linkage Disequilibrium

Linkage disequilibrium values (*r*^2^ and *p-*values) between DArT markers were calculated using TASSEL software version 5.2.18 (Bradbury et al., 2007). Minor allele loci with <0.05 frequency were filtered out to reduce biased LD estimations between pairs of loci (Gaut and Long, 2003). The *r*^2^ values for pairs of loci were plotted as a function of map distances, and LD decay (*r*^2^ < 0.19) was estimated using the average distances of marker pairs showing LD values lower than 0.19 (Whitt and Buckler, 2003).

Principal components and a kinship matrix were calculated using GAPIT statistical package in R software (Kang et al., 2008; Lipka et al., 2012). The kinship matrix was calculated based on VanRaden’s method (VanRaden, 2008). Unweighted pair group method with arithmetic mean (UPGMA) was used to cluster the wheat genotypes based on polymorphism of the 533 DArT markers with known chromosomal positions. The distribution of correlation coefficients (r^2^) between DArT markers located at different physical distances of the wheat genome was calculated to establish LD among loci.

#### Genome-Wide Association Scan

GWAS analyses were performed using the Genomic Association and Prediction Integrated Tool (GAPIT) package in R (Lipka et al., 2012). Compressed mixed linear model (CMLM) approach accounting for population parameters was used (Zhang et al., 2010). The optimum number of principal components (PCs) to be included in the final GWAS model was determined by forward model selection based on Bayesian information criterion (BIC). The following mixed model, accounting for genetic relatedness among lines was used (Zhang et al., 2010). Y = Xβ + Zu + e, where Y is the vector of observed phenotypes; β is fixed effects, including the genetic marker, population structure (Q), and the intercept; u is random additive genetic effects from multiple background QTL for individuals/lines; X and Z are the known design matrices; and e is the unobserved vector of residuals. The *p*-values from CMLM analysis were adjusted based on Benjamini-Hochberg false discovery rate controlling procedure (Benjamini and Hochberg, 1995).

## Results

### Phenotypic Variation

**FIGURE 1:**
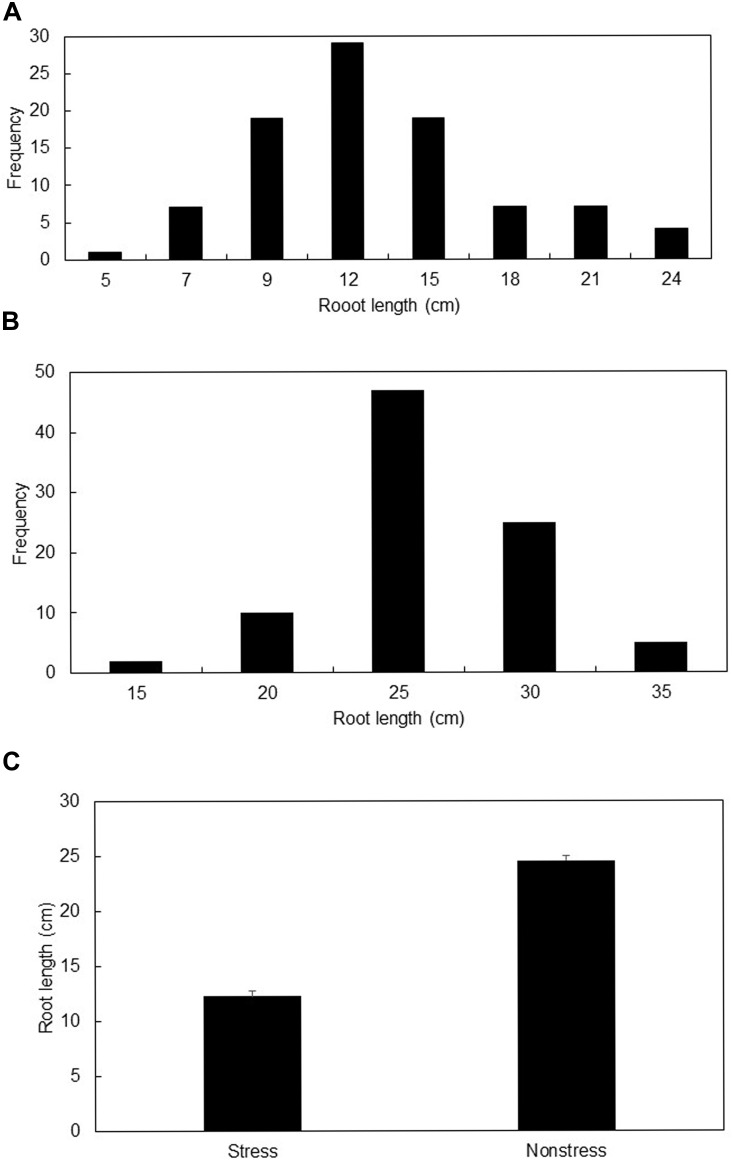
Distribution of mean root length (cm) under stress **(A)**, and non-stress **(B)** growing conditions, and the mean root length (cm) ± SE of all genotypes under stress and non-stress conditions **(C)**.

Mean root lengths ranged from 5 to 25 cm under stress, and from 13 to 32 cm under non-stress conditions (Figures 1A,B). The induced stress reduced average root length by 50.5% (Figure 1C). Genotypes “NS 63-24” and “Tr. dirk “B” (GK 12)” showed the longest roots under stress condition while genotypes “Suwwon 92” and “Holly E” were longest rooted genotypes under non-stress condition.

Analysis of variance showed significant variations for root length under the two growing conditions (Table 2). Heritability was moderate (59%) to high (73%) under non-stress and stress conditions, respectively. Genetic gain at 5% selection intensity ranged from 7.6 to 9.6 under non-stress and stress conditions, respectively (Table 2).

**Table 2:** Variance, heritability and genetic gain of root length among 91 diverse wheat genotypes grown under non-stress and stress conditions.

Growing condition	Genotypic variance	Error variance	Heritability (H^2^) (%)	Genetic gain (GA) (5%)
Non-stress	39.0^∗^	27.0	59	7.6
Stress	41.7^∗∗^	15.5	73	9.7

*^∗∗^, ^∗^indicate significant variation at p ≤ 0.01and p ≤ 0.05, respectively.*

### Genotypic Diversity and Linkage Disequilibrium

**FIGURE 2:**
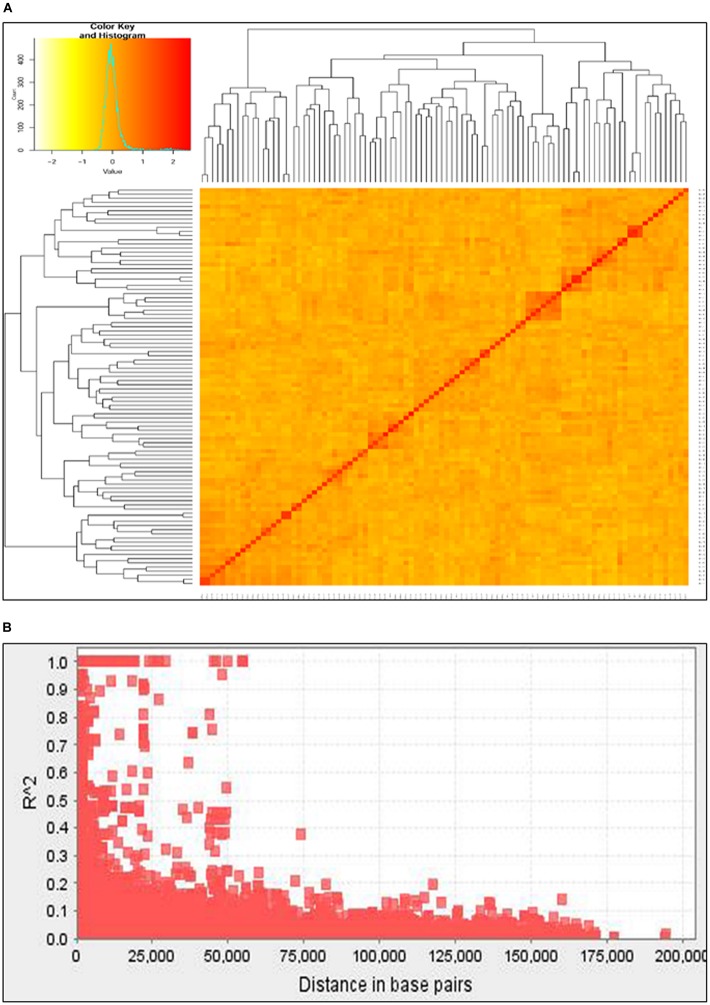
Major clusters of the 91 wheat collections based on unweighted pair group method with arithmetic mean (UPGMA) algorism **(A)** and genome-wide average LD **(B)** over genetic distance (bp).

Unweighted pair group method with arithmetic mean (UPGMA) clustering based on maximum Euclidian distance grouped the 91 wheat accessions into three major clusters (Figure 2A). Several of the principal components (PCs) also showed high eigenvalues suggesting significant diversity among the test genotypes. The first three principal components (PCs) were found optimum to fit the mixed linear model. Nearly 15% (3,877 out of 25,125) of the marker pairs showed significant LD (*p* < 0.05). A total of 196 marker pairs were in complete linkage spanning a total length of 10.45 kbp. Nearly 16% (2,285 of 14,436) of the significant LDs were found among inter chromosomal marker pairs, which were caused by factors other than physical linkage. Generally, LD value declined as the physical distance between the loci increased. The average LD value for inter chromosomal markers was 0.019 while the same was 0.069 for intra chromosomal markers. The overall average LD for all of the marker pairs showed *r*^2^ value of 0.039. The average genetic distance between markers with *p* < 0.05 was 25.39 kbp.

### Genome Wide Association Scan

**FIGURE 3:**
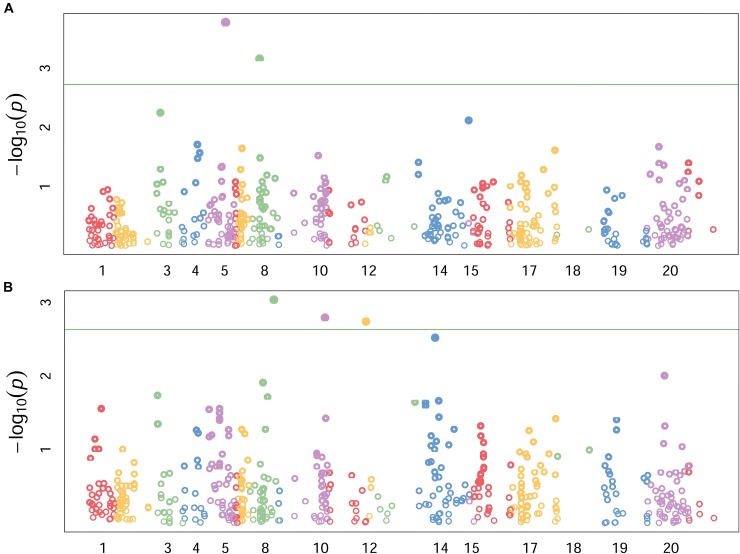
Genomic locations of significant QTLs under stress **(A)**, and non-stress **(B)** conditions. Numbers along the horizontal indicate individual chromosomes. Labels for some chromosomes with smaller number of markers are omitted (can be seen from the diffenet colors) to avoid cluttering the graph. Data pionts above the horizontal green line showed adjusted *p-*values < 0.001.

Highly significant marker trait associations were identified under the two growing conditions. The two growing conditions showed different marker-trait-associations such that none of the significant associations were common between the two water regimes. Under the stress condition, genomic regions on chromosomes 2B and 3B showed the highest peaks with *p*-values of 3E-04 and 1.1E-3, explaining 17 and 14% of phenotypic variation, respectively (Table 3 and Figure 3A). The DArT markers *wPt6278* (2B) and *wPt1159* (3B) were significantly associated with root length under stress condition. Similarly under non-stress condition, markers *wPt0021* (3B), *wPt4487* (4A) and *wPt8890* (5B) showed significant association with root length, explaining 22%, 22 and 19% of phenotypic variation, respectively (Figure 3B). The minor allele frequency (MAF) of DArT markers ranged from 0.09 to 0.43 while the smallest *p*-value was 3E-4. It was interesting to note that chromosome 3B harbored two loci significantly associated with root length each explaining 14 and 22% of phenotypic variation, under stress and non-stress conditions, respectively (Table 3).

**Table 3:** Significant root length QTLs, associated DArT markers, their chromosomal positions, and level of phenotypic variation (r^2^) explained by each of the QTLs.

Condition	Marker name	Chromosome	Position	MAF	R^2^ (%)	*p*	*q*
Stress	*wPt6278*	2B	83.9	0.08	17	3E-04	1E-03
	*wPt1159*	3B	44.4	0.06	14	1.1E-03	2E-03
Non-stress	*wPt0021*	3B	96.64	0.11	22	5E-04	1E-03
	*wPt4487*	4A	174.62	0.43	22	5E-04	2E-03
	*wPt8890*	5B	77.95	0.09	19	1.6E-03	3E-03

*MAF, minor allele frequency; p, level of significance without adjustment; q, adjusted level of significance based on Benjamini-Hochberg false discovery rate controlling procedure.*

## Discussion

### Marker Trait Association

Identification and mapping of significant association between molecular markers and various traits of interest has been the focus of modern breeding programs. The present study was one of similar efforts aimed to identify QTLs that control root length under early stage water stress and normal growing conditions. A total of five DArT markers were found significantly associated with root length under the two water regimes (Table 3). All of the significant associations reported in this study were based on adjusted *p*-values using Benjamini-Hochberg false discovery rate control (Benjamini and Hochberg, 1995). The adjusted *p*-values (indicated as *q*-values on Table 3) were at least 10 times higher than unadjusted *p*-values, which as a result has lowered the number of significant associations reported. The B genome of wheat harbored both of the significant QTLs identified under water stress condition (Table 3). Water stress resistance QTL on chromosome 3B was consistently mapped across three populations in our previous studies (Ayalew et al., 2017, 2018). Pattern of marker trait associations in the two growing conditions were quite different that none of the significant associations were common between the two water regimes even though chromosome 3B had two significant QTLs, one for each growing condition located 52 kbp apart. This might be due to the differential gene expression patterns triggered by the induced stress, which is in agreement with Bac-Molenaar et al. (2015).

On the other hand, DArT markers wPt0021 (3B), wPt4487 (4A) and wPt8890 (5B) were significantly associated with root length under non-stress growing condition. Similar previous studies reported significant early vigor QTLs located on chromosomes 3B and 4A, which were in agreement with the present findings (Bai et al., 2013; Ayalew et al., 2017, 2018). These QTLs can be incorporated into breeding programs to enhance early crop establishment and high biomass and grain yield production. The marker trait associations in this study tended to be on the B genome while none were on D genome which could be partly due to the scant marker density in the D genome. The B genome of wheat showed the highest frequency of significant associations in both water regimes indicating its role in controlling early root growth and water stress resistance.

### Linkage Disequilibrium and Genetic Structure

Thorough understanding of the genetic structure and linkage disequilibrium is vital for successful GWAS. Unweighted pair group method with arithmetic mean (UPGMA) clustering resulted in three major clusters (Figure 2A). This same population (the 91 lines included in this study plus three more genotypes) was reported to have two clusters (Neumann et al., 2011). However, Neumann et al. (2011) used only 219 of the available markers for the structure analysis which might be the reason for the fewer clusters (two) than in the present analysis (three). Principal component analysis (PCA) also showed large genetic diversity in genotypes calling for the use of mixed linear model to account for population structure and relatedness (Zhang et al., 2010).

Neumann et al. (2011) have published a detailed LD analysis of this population elsewhere using 525. The present analysis found significant LD (*p* < 0.05) in 3,877 of 25,125 (15.4%) inter chromosomal marker pairs with an average *r*^2^ value of 0.25 which was comparable (0.26) with the aforementioned publication. Comparable proportion of significant LD values (14.9%) were previously reported in wheat (Neumann et al., 2011; Dreisigacker et al., 2012; Edae et al., 2014). The average genetic distance between markers with *r*^2^> 0.1 was 15 kbp. Average LD decay for all chromosomes was estimated at approximately 35 kbp, with *r*^2^ cut-off value set to 0.25 (Figure 2B). This relatively long LD might be caused by inbreeding which limits the number of heterozygotes and the number of effective recombination rates leading to correlated genetic polymorphisms, hence long physical LD (Gaut and Long, 2003). Breeding and selection, population stratification and relatedness, genetic drift and genetic bottlenecks were reported to be among the main factors that could cause LD among non-collinear markers (Ohta, 1982; Chao et al., 2010).

### Phenotypic Variation and Potential for Genetic Improvement

As evidenced in this study early stage water stress can inflict up to 50% reduction in crop root length. Comparable reductions (40–54%) in root length were previously reported on segregating populations and diverse wheat collections (Ayalew et al., 2015, 2016a). Water stress can induce signals in different genotypes differently. Some genotypes respond by halting growth while others keep on normal physiology by defying the stress. This growth differential was observed in changes in the ranking of genotypes across the two water regimes in this study. This differential response/ranking of genotypes, which was also observed in previous experiments (Ayalew et al., 2015, 2016a,b), indicated the validity of selecting stress tolerant genotypes under the target environment (Blum, 2011). Previous findings reported the complex and dynamic nature of plant growth highlighting the different roles played by specific genes at specific growth stages (Blum, 2011; Bac-Molenaar et al., 2015). Broad sense heritability was moderate to high (59–73%) in this study, which indicated the possibility of successful root length improvement using diverse germplasm.

## Conclusion

In conclusion, the B genome of hexaploid wheat harbored two major QTLs for root length under stress condition. The importance of the B genome for early root vigor is also indicated by the presence of two root length QTLs under non-stress condition in chromosomes 3B and 5B. Genetic improvement programs for contrasting moisture conditions need to be targeted separately due to qualitative QTL by environment interactions. The identified markers can potentially be validated and incorporated into MAS programs for root length improvement.

## Author Contributions

HA and GY conceived the project. HA carried out the experiment and wrote the draft manuscript. GY, HL, AB, BK, and CL reviewed and edited the manuscript. All authors have read and approved this manuscript for submission.

## Conflict of Interest Statement

The authors declare that the research was conducted in the absence of any commercial or financial relationships that could be construed as a potential conflict of interest.

## Footnotes

1 http://www.triticarte.com.au/
