# Supplementary material for: Genome-Wide Association Mapping of Major Root Length QTLs Under PEG Induced Water Stress in Wheat
Source: Front Plant Sci. 2018 Nov 29;9:1759. doi: 10.3389/fpls.2018.01759 (PMC6281995; doi:10.3389/fpls.2018.01759)
Supplement: Supplementary file 1 [file Data_Sheet_1.pdf]

Genome-wide association mapping of major root length QTLs under PEG induced water stress in wheat.

Habtamu Ayalew[1, 2], Hui Liu[1], Andreas Börner[3], Borislav Kobiljski[4], Chunji Liu[5], Guijun Yan[1]*
[1]School of Agriculture and Environment, Faculty of Science and The UWA Institute of Agriculture, The University of Western Australia, Crawley, WA, Australia.[2]Noble Research Institute LLC, Ardmore, OK, USA. [3]Genebank Department, Leibniz Institute of Plant Genetics and Crop Plant Research (IPK), Gatersleben, Germany. [4]Institute of Field and Vegetable Crops, Novi Sad, Serbia. [5]CSIRO Agriculture Flagship, QLD, Australia
*Correspondence:
Prof Guijun Yan: guijun.yan@uwa.edu.au

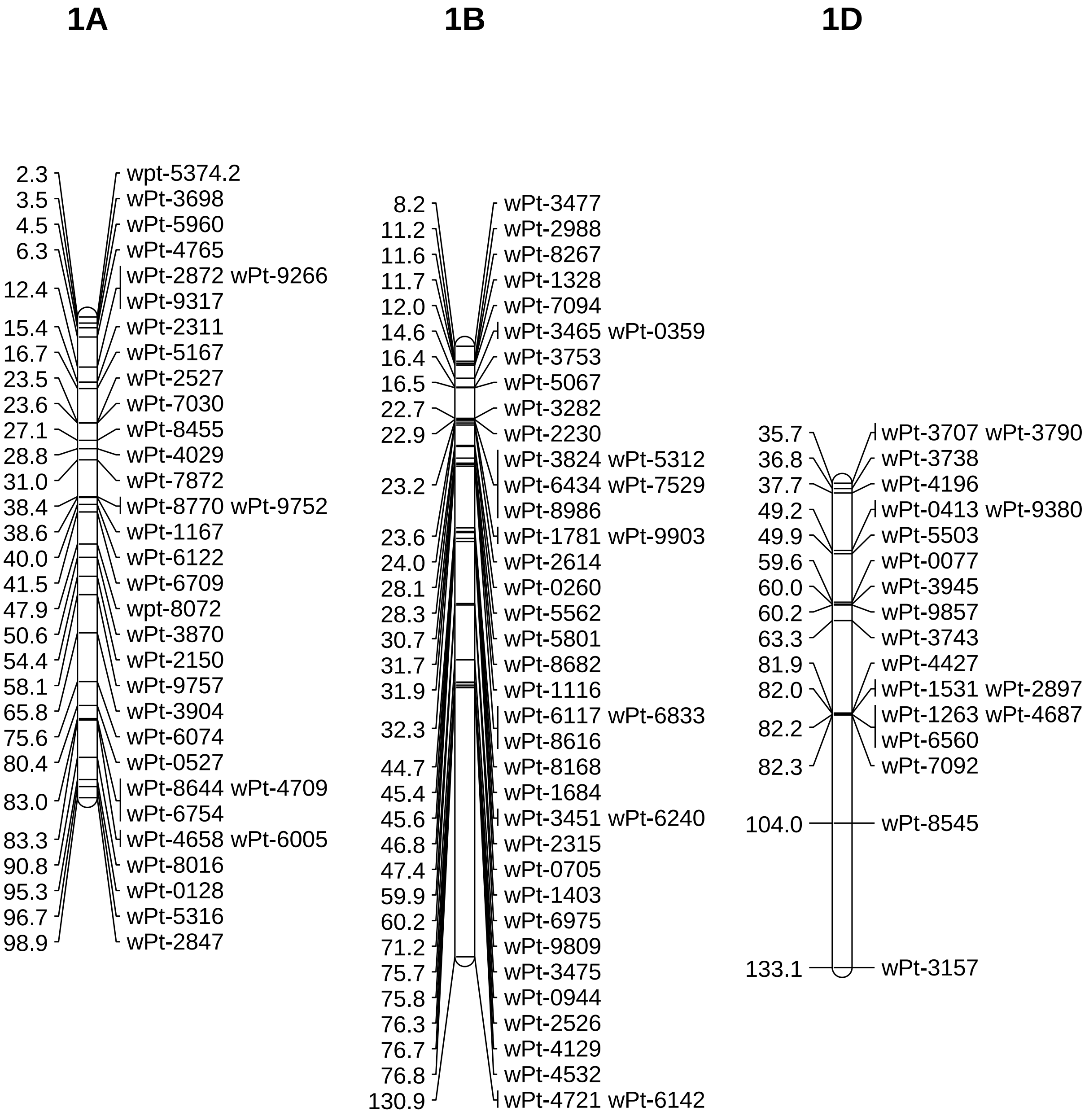

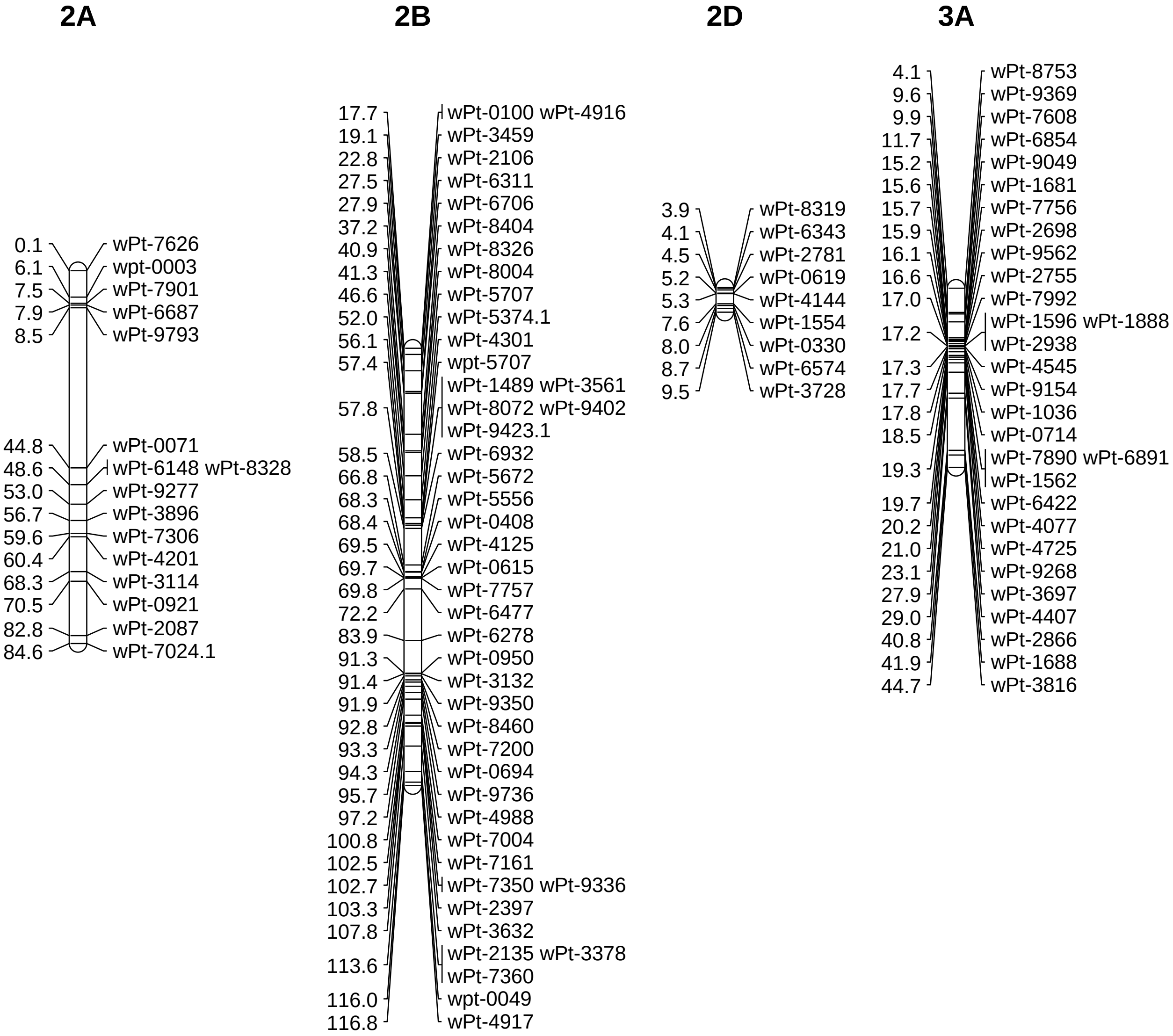
2A
0.1 wPt-7626
6.1 wpt-0003
7.5 wPt-7901
7.9 wPt-6687
8.5 wPt-9793
44.8 wPt-0071
48.6 wPt-6148 wPt-8328
53.0 wPt-9277
56.7 wPt-3896
59.6 wPt-7306
60.4 wPt-4201
68.3 wPt-3114
70.5 wPt-0921
82.8 wPt-2087
84.6 wPt-7024.1
2B
17.7 wPt-0100 wPt-4916
19.1 wPt-3459
22.8 wPt-2106
27.5 wPt-6311
27.9 wPt-6706
37.2 wPt-8404
40.9 wPt-8326
41.3 wPt-8004
46.6 wPt-5707
52.0 wPt-5374.1
56.1 wPt-4301
57.4 wpt-5707
57.8 wPt-1489 wPt-3561 wPt-8072 wPt-9402 wPt-9423.1
58.5 wPt-6932
66.8 wPt-5672
68.3 wPt-5556
68.4 wPt-0408
69.5 wPt-4125
69.7 wPt-0615
69.8 wPt-7757
72.2 wPt-6477
83.9 wPt-6278
91.3 wPt-0950
91.4 wPt-3132
91.9 wPt-9350
92.8 wPt-8460
93.3 wPt-7200
94.3 wPt-0694
95.7 wPt-9736
97.2 wPt-4988
100.8 wPt-7004
102.5 wPt-7161
102.7 wPt-7350 wPt-9336
103.3 wPt-2397
107.8 wPt-3632
113.6 wPt-2135 wPt-3378 wPt-7360
116.0 wpt-0049
116.8 wPt-4917
2D
3.9 wPt-8319
4.1 wPt-6343
4.5 wPt-2781
5.2 wPt-0619
5.3 wPt-4144
7.6 wPt-1554
8.0 wPt-0330
8.7 wPt-6574
9.5 wPt-3728
3A
4.1 wPt-8753
9.6 wPt-9369
9.9 wPt-7608
11.7 wPt-6854
15.2 wPt-9049
15.6 wPt-1681
15.7 wPt-7756
15.9 wPt-2698
16.1 wPt-9562
16.6 wPt-2755
17.0 wPt-7992
17.2 wPt-1596 wPt-1888 wPt-2938
17.3 wPt-4545
17.7 wPt-9154
17.8 wPt-1036
18.5 wPt-0714
19.3 wPt-7890 wPt-6891 wPt-1562
19.7 wPt-6422
20.2 wPt-4077
21.0 wPt-4725
23.1 wPt-9268
27.9 wPt-3697
29.0 wPt-4407
40.8 wPt-2866
41.9 wPt-1688
44.7 wPt-3816

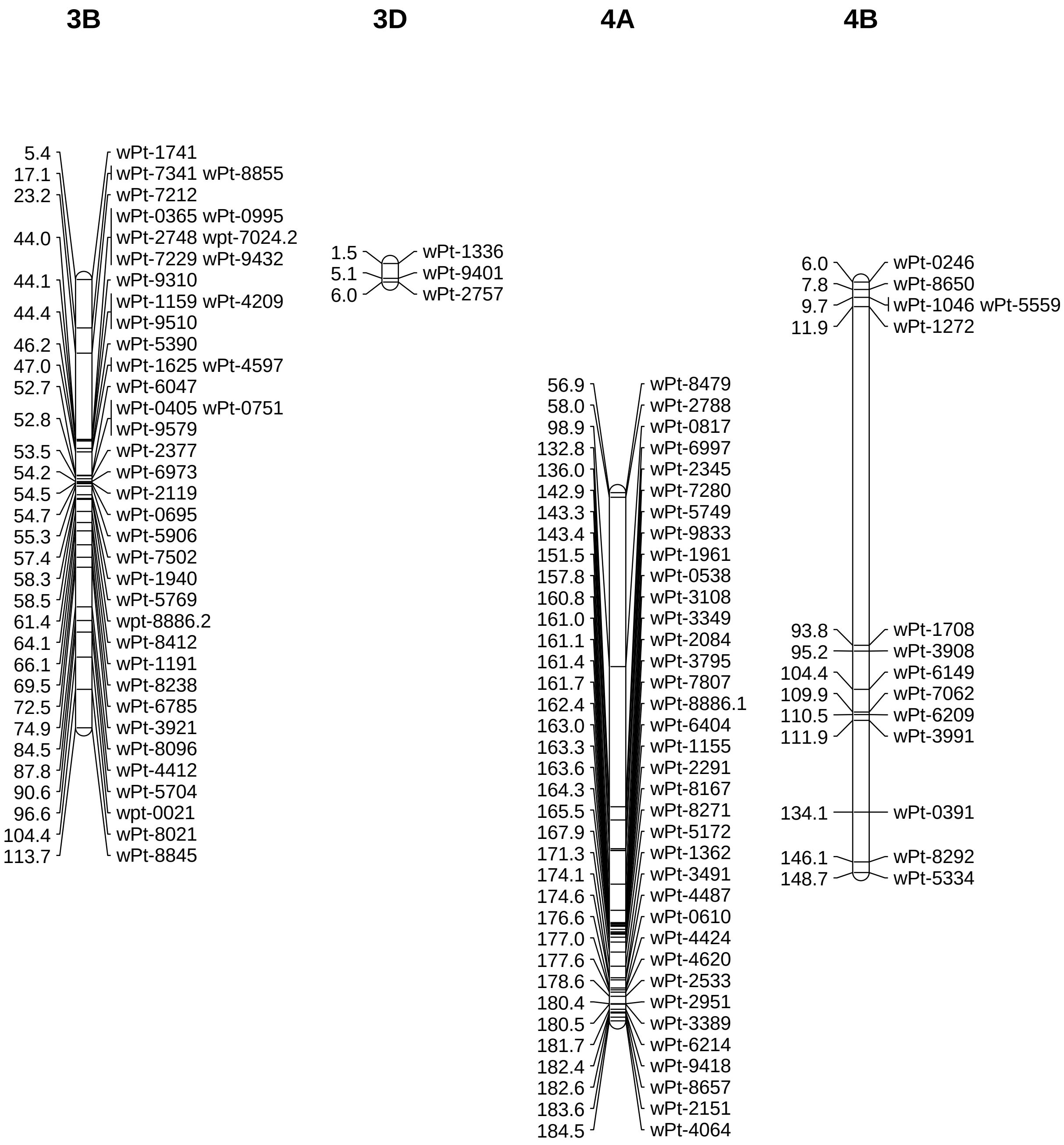
3B
5.4 wPt-1741
17.1 wPt-7341 wPt-8855
23.2 wPt-7212
wPt-0365 wPt-0995
44.0 wPt-2748 wpt-7024.2
wPt-7229 wPt-9432
44.1 wPt-9310
wPt-1159 wPt-4209
44.4 wPt-9510
46.2 wPt-5390
47.0 wPt-1625 wPt-4597
52.7 wPt-6047
52.8 wPt-0405 wPt-0751
wPt-9579
53.5 wPt-2377
54.2 wPt-6973
54.5 wPt-2119
54.7 wPt-0695
55.3 wPt-5906
57.4 wPt-7502
58.3 wPt-1940
58.5 wPt-5769
61.4 wpt-8886.2
64.1 wPt-8412
66.1 wPt-1191
69.5 wPt-8238
72.5 wPt-6785
74.9 wPt-3921
84.5 wPt-8096
87.8 wPt-4412
90.6 wPt-5704
96.6 wpt-0021
104.4 wPt-8021
113.7 wPt-8845
3D
1.5 wPt-1336
5.1 wPt-9401
6.0 wPt-2757
4A
56.9 wPt-8479
58.0 wPt-2788
98.9 wPt-0817
132.8 wPt-6997
136.0 wPt-2345
142.9 wPt-7280
143.3 wPt-5749
143.4 wPt-9833
151.5 wPt-1961
157.8 wPt-0538
160.8 wPt-3108
161.0 wPt-3349
161.1 wPt-2084
161.4 wPt-3795
161.7 wPt-7807
162.4 wPt-8886.1
163.0 wPt-6404
163.3 wPt-1155
163.6 wPt-2291
164.3 wPt-8167
165.5 wPt-8271
167.9 wPt-5172
171.3 wPt-1362
174.1 wPt-3491
174.6 wPt-4487
176.6 wPt-0610
177.0 wPt-4424
177.6 wPt-4620
178.6 wPt-2533
180.4 wPt-2951
180.5 wPt-3389
181.7 wPt-6214
182.4 wPt-9418
182.6 wPt-8657
183.6 wPt-2151
184.5 wPt-4064
4B
6.0 wPt-0246
7.8 wPt-8650
9.7 wPt-1046 wPt-5559
11.9 wPt-1272
93.8 wPt-1708
95.2 wPt-3908
104.4 wPt-6149
109.9 wPt-7062
110.5 wPt-6209
111.9 wPt-3991
134.1 wPt-0391
146.1 wPt-8292
148.7 wPt-5334

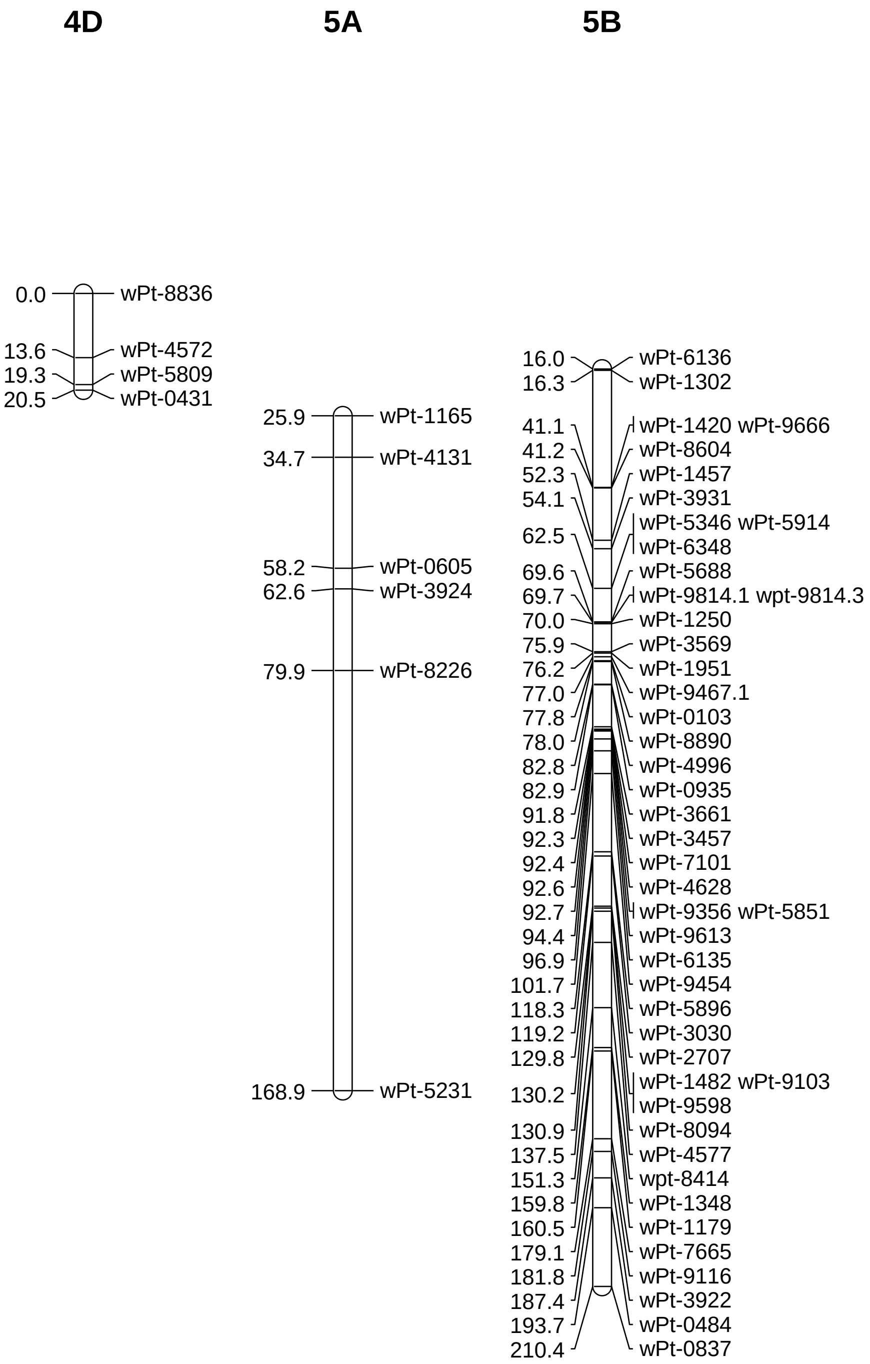

Ayalew et al. 2018

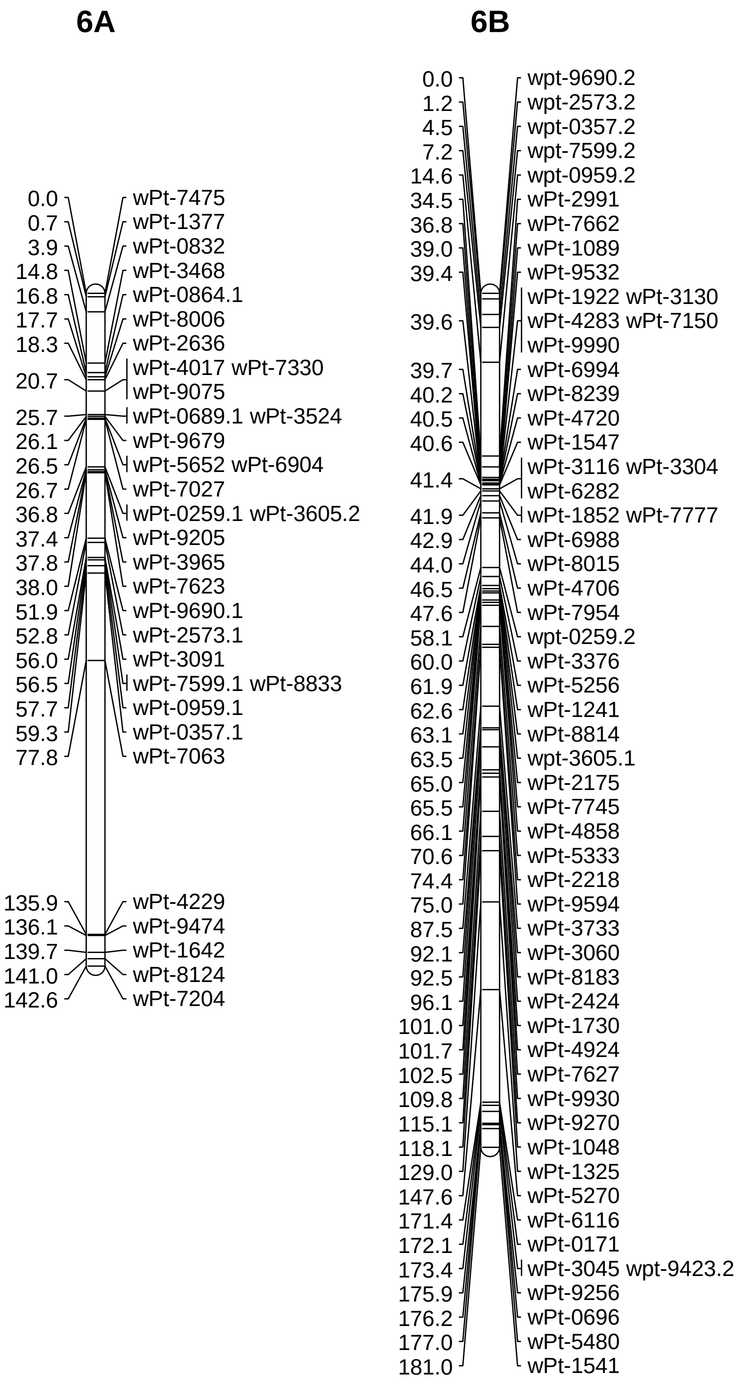

Ayalew et al. 2018

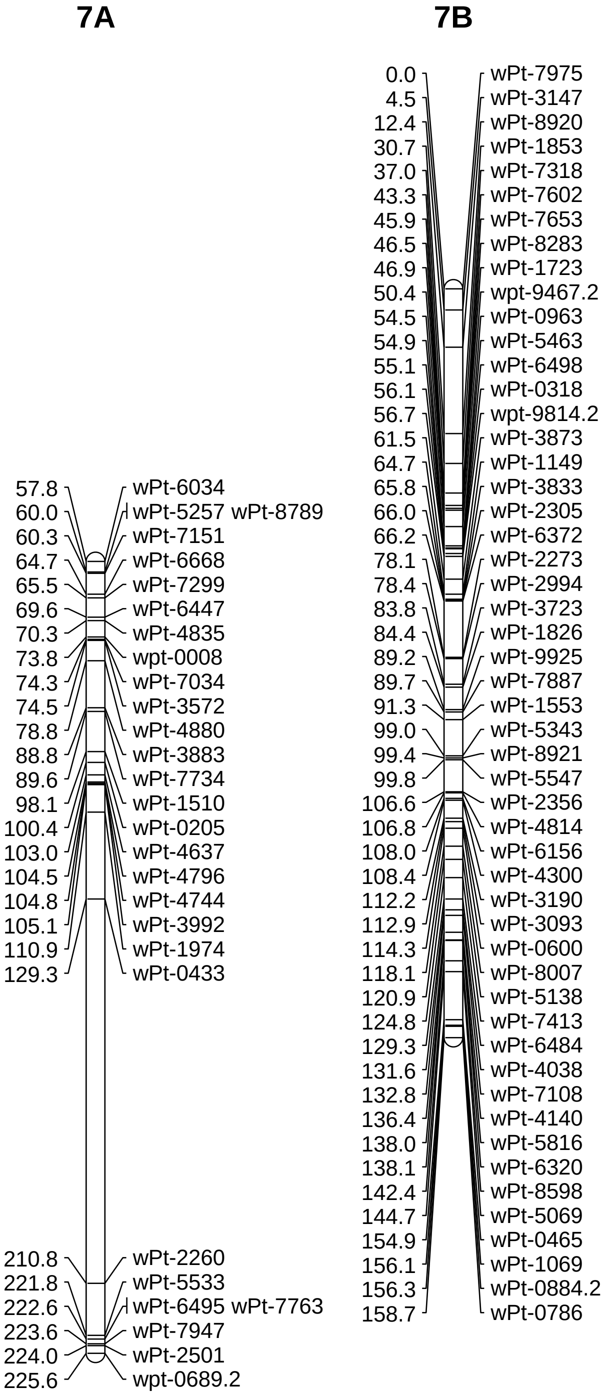

Supplementary Figure 1. Linkage map of 533 DArT markers based on CIMMYT integrated map (https://wheat.pw.usda.gov/cgi-bin/graingenes/report.cgi?class=mapdata;name=Wheat%2C%20CIMMYT%20Integrated;show=map)
